# A Genome-Wide Methylation Approach Identifies a New Hypermethylated Gene Panel in Ulcerative Colitis

**DOI:** 10.3390/ijms17081291

**Published:** 2016-08-09

**Authors:** Keunsoo Kang, Jin-Han Bae, Kyudong Han, Eun Soo Kim, Tae-Oh Kim, Joo Mi Yi

**Affiliations:** 1 Department of Microbiology, Dankook University, Cheonan 31116, Korea; kangk1204@gmail.com; 2 Research Center, Dongnam Institute of Radiological & Medical Sciences (DIRAMS), Busan 46033, Korea; 82jinhan@pusan.ac.kr; 3 Department of Nanobiomedical Science Global Research Center for Regenerative Medicine, Dankook University, Cheonan 31116, Korea; kyudong.han@gmail.com; 4 DKU-Theragen Institute for NGS Analysis (DTiNa), Dankook University; Cheonan 31116, Korea; 5 Division of Gastroenterology, Department of Internal Medicine, Kyungpook National University School of Medicine, Daegu 41931, Korea; dandy813@hanmail.net; 6 Department of Internal Medicine, Haeundae Paik Hospital, Inje University College of Medicine, Busan 48108, Korea

**Keywords:** DNA methylation profile, promoter hypermethylation, ulcerative colitis, biomarker

## Abstract

The cause of inflammatory bowel disease (IBD) is still unknown, but there is growing evidence that environmental factors such as epigenetic changes can contribute to the disease etiology. The aim of this study was to identify newly hypermethylated genes in ulcerative colitis (UC) using a genome-wide DNA methylation approach. Using an Infinium HumanMethylation450 BeadChip array, we screened the DNA methylation changes in three normal colon controls and eight UC patients. Using these methylation profiles, 48 probes associated with CpG promoter methylation showed differential hypermethylation between UC patients and normal controls. Technical validations for methylation analyses in a larger series of UC patients (*n* = 79) were performed by methylation-specific PCR (MSP) and bisulfite sequencing analysis. We finally found that three genes (*FAM217B*, *KIAA1614* and *RIBC2*) that were significantly elevating the promoter methylation levels in UC compared to normal controls. Interestingly, we confirmed that three genes were transcriptionally silenced in UC patient samples by qRT-PCR, suggesting that their silencing is correlated with the promoter hypermethylation. Pathway analyses were performed using GO and KEGG databases with differentially hypermethylated genes in UC. Our results highlight that aberrant hypermethylation was identified in UC patients which can be a potential biomarker for detecting UC. Moreover, pathway-enriched hypermethylated genes are possibly implicating important cellular function in the pathogenesis of UC. Overall, this study describes a newly hypermethylated gene panel in UC patients and provides new clinical information that can be used for the diagnosis and therapeutic treatment of IBD.

## 1. Introduction

Inflammatory bowel disease (IBD) is a chronic, relapsing, remitting, and/or continuously active disease of the gastrointestinal tract that is occasionally associated with extra-intestinal manifestations [1]. IBD can be classified into Crohn’s disease (CD) and ulcerative colitis (UC). Each type exhibits distinct etiologies and clinical features [2]. Although the exact etiology of IBD remains unknown, numerous clinical and experimental reports including genome-wide association studies have suggested that IBD is a consequence of the complex, dysregulated interplay between genetic predispositions, environmental factors, and microbial compositions in the intestine [3,4,5,6]. These genetic studies have suggested that genetic discoveries can help understand the susceptibility to IBD, although environmental factors are also important in its pathogenesis. Regarding the interaction between the environment and genome, epigenetic mechanisms may contribute to the pathogenesis of IBD.

Epigenetics is a widely investigated field of cancer biology. It deals with the regulation of gene expression, primarily through DNA methylation, miRNA or histone modifications such as acetylation, methylation, phosphorylation, or ubiquitination. DNA methylation is the most extensively studied epigenetic modification in mammals. Dysregulation of epigenetic processes can lead to altered gene function and malignant cellular transformation. During the last decade, DNA methylation has been the most studied epigenetic modification. It has been correlated with transcriptional gene silencing in human diseases, including its well established role in cancer [7]. Although numerous studies have associated DNA methylation with cancer development and dysplasia, it was only recently that DNA methylation has been implicated in the pathogenesis of IBD. To understand the relationship of hypermethylated genes with the pathogenesis and clinical aspects of IBD, most reports emphasized the studies of UC patients or IBD-associated colon cancers. However, there has been a lack of studies on genome-wide DNA methylation alterations in IBD patients.

Genome-wide DNA methylation profiling technology with the Illumina Human Methylation 450K array facilitates query of >450,000 loci within the genome and to cover 99% of RefSeq genes [8]. This improved technology permits a more powerful and comprehensive analysis of DNA methylation changes. In the present study, we used this technology to identify the locus or gene-specific DNA methylation changes in UC patients. Identification of molecular pathways with aberrant epigenetic regulation by promoter hypermethylation could provide novel methylation biomarkers for UC and possibly suggest new interventions for therapeutic treatments for IBD disease.

## 2. Results

### 2.1. Genome-Wide DNA Methylation Changes in UC Patients

Promoter CpG island hypermethylation of tumor suppressor genes is a common hallmark of various human cancers [9]. This epigenetic event can affect all cellular signaling pathways that contribute to tumorigenesis. The biological and clinical importance of hypermethylation-mediated gene silencing has been extensively studied in other human diseases such as developmental diseases, and recently in inflammatory diseases [10,11]. We have previously reported that several genes that are specifically hypermethylated in colon cancer are also hypermethylated in UC patients [12]. Although this observation suggested that DNA methylation might be useful as a diagnostic or prognostic marker for patients with UC, no IBD direct factors altered by epigenetic regulation have been reported so far.

**Figure 1:**
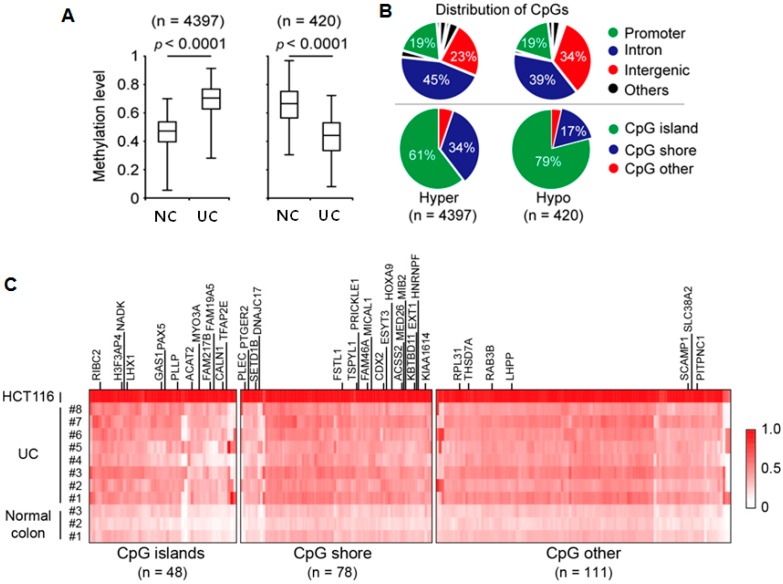
Genome-wide DNA methylation profiles of ulcerative colitis (UC) patients. (**A**) Comparison of genome-wide DNA methylation levels between UC tissues and normal control. 4397 probes showed significantly increased methylation level (hypermethylation) in UC tissues than normal colon (NC). However, 420 probes showed decreased methylation level (hypomethylation) in UC tissues than NC. *p*-values were calculated by Mann-Whitney U test; (**B**) Distributions of CpGs according to genome annotation. Most altered CpG sites in UC are located in CpG islands near the promoter regions; (**C**) Unsupervised clustering analysis of the normal colon (NC) and UC patient tissues (UC). The color gradient from white to red displays the β-values ranging from 0 (unmethylated) to 1 (fully methylated).

To identify novel DNA methylation markers mostly occurring in the CpG island, which can be used to distinguish UC patients and normal controls, we screened genome-wide methylation patterns of colon samples from control subjects (*n* = 3) and UC patients (*n* = 8) using the Human Methylation 450K BeadChip array (Illumina). The probe call rate was >99% for all samples and 454,215 CpG sites out of 485,577 were included in the analysis. The methylation level at the CpG site is measured by means of a continuous variable β-value. A value of 0 indicates a fully unmethylated site while a value of 1 indicates a fully methylated site. After eliminating unreliable probes (difference of β-values < 0.2), we identified a large number of probes, which showed significant changes in DNA methylation between UC patients and normal colons (Figure 1A). Among the probes, the majority of them (*n* = 4397) were hypermethylated, while 420 probes were hypomethylated in UC samples when compared with normal colon samples. Notably, most of the differentially methylated CpG sites were located in the CpG islands (Figure 1B). According to gene annotation, intron, intergenic and promoter regions were the major parts of the genome that contained susceptible CpG sites specific to UC patients. To identify relevant increasing methylation level in UC patients, the identified probes were further filtered with a strict criteria (>1.7-fold change of β-values in UC patients compared to normal colon). Unsupervised hierarchical cluster analyses distinguished normal colon and UC patient tissues. A total of 237 hypermethylated probes were identified that represented hypermethylation patterns in UC patients when compare with normal colons (Figure 1C). We used HCT116 colon cancer cells as a positive control for global methylation analysis. As expected, all of the candidates were hypermethylated in HCT116 colon cancer cells. Collectively, we identified 237 CpG sites that were significantly hypermethylated in UC patients compared to a normal colon.

### 2.2. Validation of Selected Candidate Genes in UC Patient Samples

**Figure 2:**
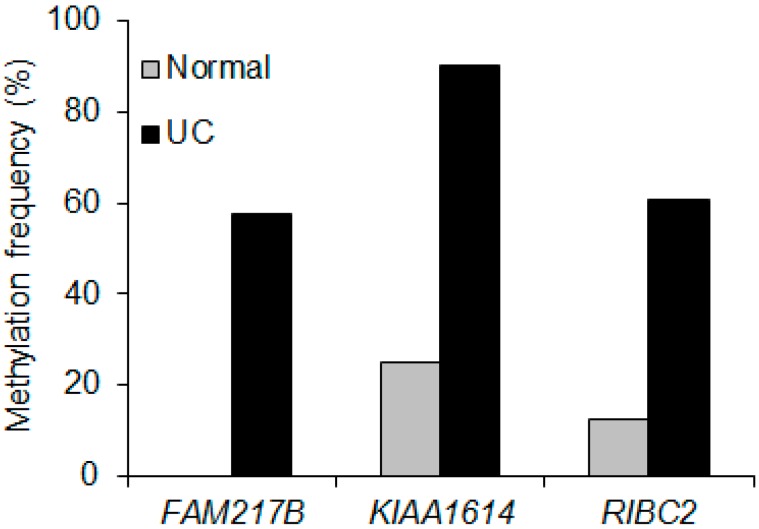
DNA methylation frequencies of three best candidate genes (*FAM217B*, *KIA1614* and *RIBC2*) in UC samples (*n* = 79) and normal colon (NC) (*n* = 8).

As shown in Figure 1C, 48 out of 237 probes have typical CpG islands, defined as 200 bp sequences containing greater than 50% CpG dinucleotides [13], in their promoter regions, which could regulate their transcriptional gene expression. The gene list is summarized in Table S1. To identify newly hypermethylated genes in UC patients, we performed the validation for these genes using MSP and bisulfite sequencing analyses in a large series of UC samples (*n* = 79) (Table 1). We designed primers for methylation analysis for all 45 genes (Table S2). Since our methylation profile showed candidate genes were relatively hypermethylated pattern in HCT116 cells, we therefore first tested the methylation levels of these genes in HCT116 cells by MSP (Figure 1C). We confirmed that 26 out of 45 genes were hypermethylated in HCT116 cells and the rest of them were illuminated. To filter down genes are increasing methylation level in UC compare to normal controls, the following experimental validation criteria was based on our previous studies were used [14,15]: (i) the presence of gene expression in normal colon tissue; (ii) low or no methylation in normal colon tissues; and (iii) the presence of methylation in primary UC samples. The three best candidate genes, including RIB43A domain with coiled-coiles 2 (*RIBC2*), family with sequence similarity 217 mermber B (*FAM217B*) and *KIAA1614*, fulfilled the above criteria on our validation criteria. We performed massive MSP analysis for this validation and found that all three genes, *KIAA1614*, *RIBC2*, and *FAM217B*, showed 91%, 64% and 62% methylation in the UC samples, respectively (*n* = 79) (Figure 2).

**Figure 3:**
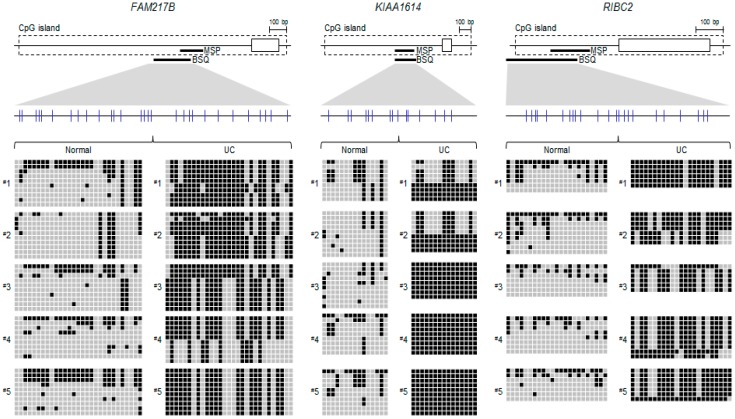
Bisulfite sequencing analyses of the CpG island in *FAM217B*, *KIA1614*, and *RIBC2* gene promoter regions. A schematic representation of each gene CpG island (box). The regions analyzed using methylation-specific PCR (MSP) and bisulfite sequencing are indicated by black bars below the CpG island. Individual CpG sites are indicated as vertical lines. Representative bisulfite sequencing analyses were performed for all three genes in representative UC patient samples (*n* = 5) and controls (*n* = 5). Each box represents a CpG dinucleotide. Black boxes represent methylated cytosines and gray boxes represent unmethylated cytosines.

**Figure 4:**
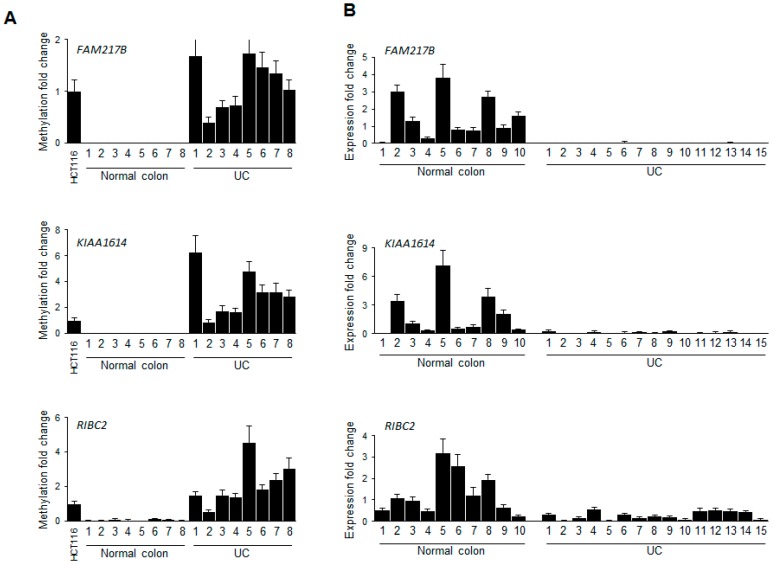
Correlation between promoter hypermethylation and transcriptional silencing in UC patient tissues and in controls. (**A**) Quantitative MSP and (**B**) RT-PCR analyses of *FAM217B*, *KIAA1614* and *RIBC2* genes in selective UC patient samples and controls. HCT116 cells were used as a positive control for methylation levels. All quantitative methylation levels were normalized by the *Alu* element. Human *GAPDH* was used for expression normalization. Statistical significance (*p* < 0.001) for all three genes is shown between UC patient tissue samples and controls.

We next confirmed the CpG island methylation level of the three genes via bisulfite sequencing analysis in representative UC samples along with normal colon tissues (Figure 3). Notably, all three genes were more densely methylated in UC samples compared to normal colon tissues. Moreover, we also verified the methylation levels of the three genes in representative UC patient samples using quantitative MSP analysis (Figure 4A), suggesting that the methylation levels are increased in UC samples (*n* = 8) compare to normal colon tissues (*n* = 8). Overall, these results strongly suggested that the final candidate genes showed increasing DNA methylation levels in UC samples compare with normal controls.

Next, we investigated whether hypermethylation of these genes correlated with downregulation of transcriptional gene expression levels in UC samples. Quantitative RT-PCR (qRT-PCR) showed that all three genes were significantly downregulated in representative UC samples (*n* = 15) when compared with normal control samples (*n* = 10) (Figure 4B). Combining the results of methylation and transcriptional gene expression patterns suggested that hypermethylation of the candidate gene panel was correlated to their transcriptional repression in UC samples.

### 2.3. Functional Implications of the UC Hypermethylated Gene Panel

**Figure 5:**
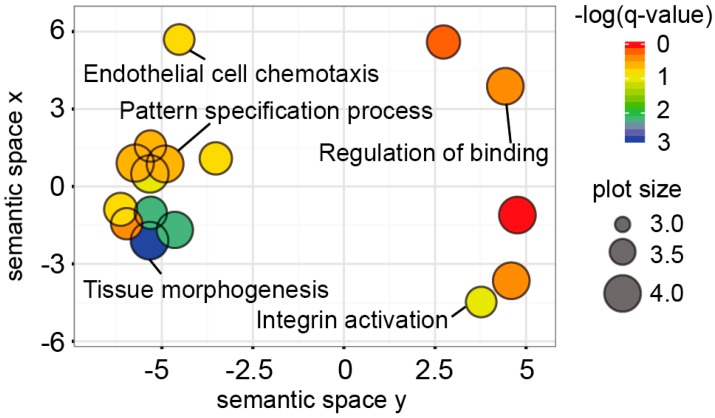
Gene ontology analysis and functional implication of hypermethylated genes in UC. Gene ontology (GO) analysis was performed using GeneMANIA with nearby genes around 48 hypermethylated CpG sites which coincides with CpG islands. The x and y axis represent arbitrary numbers. Similar terms tend to be clustered in the plot. The size of circle depicts whether a given term is a more general GO term (**larger**) or a more specific one (**smaller**).

It has been suggested that abnormal signaling pathways play an important role in the inflammatory processes leading to dysregulation of the inflammatory responses that are involved in the pathogenesis of IBD [16]. In the present study, we investigated how these hypermethylated genes were associated with the cellular pathway network. Using our hypermethylated gene panel, we performed gene ontology analysis with well curated databases (e.g., KEGG) including a variety biological processes, molecular functions, and cellular components. Redundant terms were removed using the REVIGO application [17]. The most significant pathway affected by the hypermethylated genes was related to tissue morphogenesis (Figure 5). The *ANKRD1*, *HES1*, *HGF*, *LHX1*, *LRP5*, *NRP1*, *PRICKLE1*, *PROX1*, *SOX11*, *SRF*, *TGFBR3* and *TPM1* genes were associated with the pathway. In addition, integrin activation-related genes including *CXCL13*, *PIEZO1* and *PTGER4* became hypermethylated in UC. Further investigations of those genes are required to unveil the relationship between the hypermethylated genes and ulcerative colitis (Figure 5).

## 3. Discussion

The etiology of UC sometimes involves changes in genetic loci, which causes over 16% of the cases of this disease [5]. Epigenetic modifications involving other factors may explain the remaining causes or risk of the disease [18,19,20]. There is increasing evidence that environmental factors regulate the epigenetic alterations and therefore contribute to disease susceptibility, manifestation, and progression [21]. Although multiple studies have found aberrant promoter methylation of a number of genes in human patients with UC [22], IBD-related DNA methylation studies have emphasized the development of colon cancer associated with IBD [14,23,24]. To specifically identify hypermethylated genes or loci associated with UC, recent technological advances have facilitated the assessment of global DNA methylation patterns of patients with UC. Very recently, 25 genes associated with inflammation have been differenetially methylated during inflammation process, which implicated in cancer development in UC [25]. However, very few epigenetic factors regulated by hypermethylation in a large cohort of UC patients have been reported correlated to the transcriptional repression.

Here, we analyzed genome-wide methylation profiles of patients with UC using an Illumina Human Methylation 450K array. After identifying a subset of hypermethylated genes (*n* = 45) with a strict criteria of >1.7-fold β-values in patients with UC, we validated the methylation levels of these genes using multiple methylation analyses and correlations with transcriptional expressions in primary samples of UC patients by qRT-PCR. We finally identified three genes (*FAM217B*, *KIAA1614* and *RIBC2*) that were hypermethylated in UC patient samples associated with transcriptional repression. The three genes were also strongly hypermethylated in most colon cancer cell lines we tested (Figure S1). None of these three genes have been previously shown to be hypermethylated in human diseases including UC or cancer, and a biological function in human diseases. To the best of our knowledge, this is the first study to report that the expression of these genes is regulated by promoter DNA hypermethylation in UC. Thus, the epigenetic effects by DNA hypermethylation could be useful for prognosis of UC as a molecular biomarker. Although we used a small set of UC patient samples for screening with a methylation array, we still provided promising candidate genes that showed frequently hypermethylated and significantly increasing the methylation level in UC patient samples.

Epigenetic regulation of tumor suppressor genes by promoter CpG hypermethylation is well established as an important mechanism for gene inactivation [26]. In addition, epigenetic alterations have become established in recent years as one of the most important molecular signatures of human diseases. Our candidate genes can be linked to disruption of many cellular pathways involving DNA repair, apoptosis and the cell cycle, especially in cancer. Signaling pathway analyses with our candidate genes implicated hypermethylated genes in patients with UC, which might contribute to identifying key cellular pathways leading to inflammatory diseases (Figure 5). Taken together, these results provide the basis for using the methylation profile of UC patients to obtain a better understanding of the molecular pathways in the pathogenesis of UC.

Numerous potential clinical applications of epigenetics for diagnostic and therapeutic applications have been reported [27]. DNA methylation is an attractive biomarker, because it has been related to many different clinical aspects, such as disease severity, duration, phenotype, inflammation and dysplasia. Recent advances in our understanding of IBD-associated DNA methylation provide many promising clinical applications, such as the use of molecular biomarkers for diagnosis and prognosis of the disease, as well as prediction of treatment outcomes. Karatzas et al. have observed the specific DNA methylation signature in UC and CD patients using EpiTect Methyl II Signature PCR Array profiles [28]. Other group have identified the differentially methylated locus in blood samples with CD using HumanMethylation 450 K BeadChip platform [29]. In addition, emerging epigenetic and genetic approach can be useful for improving potential application for IBD surveillance [30].

However, these applications were limited by the absence of suitable targets, because DNA methylation frequencies of many candidate genes are not high enough to be clinically used. Earlier reports have demonstrated that ESR-1 (Oestrogen receptor-1) and N-33 (tumor suppressor candidate-3) have increased their promoter methylation in patients with UC [30]. Moreover, it has recently been suggested that detecting methylation of *FOXE1* and *SYNE1* genes in colitis-associated colorectal neoplasia could be a promising biomarker [31]. However, both genes have shown lower methylation frequencies (less than 50%) in our samples (Figure S2).

In this study, we show that three novel genes are frequently methylated (>60%) in a larger validation set, strongly suggesting that this gene panel could be a valuable methylation biomarker for prognosis or diagnosis of UC patients. More extensive studies will be necessary to validate this gene panel in a larger cohort of UC patients, which could strongly support its clinical application in the treatment of IBD. Because a large number of studies have reported the feasibility of detecting promoter hypermethylation of multiple genes in serum, stool, or body fluids in a broad spectrum of tumor types [32,33,34], further studies will be necessary to confirm that our candidate genes can be useful in screening serum or stool samples of UC patients.

Although the clinical relevance of epigenetic aspects of IBD have not been thoroughly characterized, several studies have reported that epigenetic mechanisms are implicated in the pathogenesis of UC. For example, *p16INK4a* methylation was observed in regions negative for dysplasia, as well as during neoplastic progression in UC [23]. Furthermore, other studies have reported that promoter methylations of *E-cadherin*, *CDH1* and *GDNF* are more frequently detected in long-standing UC [35,36].

We therefore investigated whether our hypermethylation profile was associated with clinical outcomes of UC patients, such as disease duration, location, or other clinical features. In contrast with previous studies, our methylation profile using 45 hypermethylated genes was associated with inflammatory activity. Clustering analyses suggested that UC patients with active mucosal inflammation, as assessed by endoscopy, displayed increasing methylation levels when compared with inactive mucosal inflammation. The relationship between samples using multiimentional scaling (MDS) analysis also supports that DNA methylation profile is associated to the mucosal inflammation activity (Figure S3A). Notably, we did not identify any correlations between the hypermethylation profiles and long-standing UC samples which had been reported by previous studies. While there was no correlation between disease duration of UC patients and hypermethylated genes, UC patients with active (UC 6 and 8) and inactive (UC 1, 3 and 5) mucosal inflammation were significantly divided by hierarchical analysis using CpG promoter hypermethylated genes, suggesting that our selective hypermethylated gene profiles were clinically relevant loci during UC development (Figure S3B).

Our results showed that a global DNA methylation screening platform can identify a novel hypermethylated gene panel in patients with UC. Using various methylation analyses, the methylation status of candidate genes (*FAM217B*, *KIAA1614* and *RIBC2*) was validated in a larger panel of UC patient samples. This gene hypermethylation pattern was also correlated with transcriptional silencing in the mRNA of UC patients. We therefore propose that this novel hypermethylated gene panel could be a valuable biomarker for diagnosis and prognosis of UC patients. In addition, the integrative DNA methylation profile of UC patients was associated with disruption of cellular pathways contributing to IBD pathogenesis, suggesting the potential importance of epigenetic mechanisms involving promoter hypermethylation in the modulation of IBD. Overall, our findings may lead to the use of DNA methylation data for novel clinical applications, diagnoses and treatments for IBD.

## 4. Materials and Methods

### 4.1. Tissue Samples

Tissue samples were collected from the rectum at the time of colonoscopy. All patients had a confirmed diagnosis of UC based on their clinical symptoms, and endoscopic and pathological findings. The inflammatory status was classified as either active or inactive, based upon endoscopy. The main clinicopathological characteristics of the patients for experimental validation are described in Table 1. Normal colon samples (*n* = 8, median age: 36 years) as control were used for transcriptional expression and methylation analyses. The UC and normal colon biospecimens for this study were provided by the Keimyung Human Bio-Resource Bank (KHBB) and Inje Biobank, a member of the National Biobank of Korea, which is supported by the Ministry of Health and Welfare. This study was approved by the respective institutioal review board of the participating institutions of the National Biobank of Korea and written, informed consent was obtained for all study participants prior to data collection.

### 4.2. Ethic Statement

This study was approved by the respective institutional review board of the participating institutions of the National Biobank of Korea and written, informed consent was obtained for all study participants prior to data collection. All samples or specimen derived from the Inje Biobank were obtained with informed consent under the institutional review board (IRB)-approved protocols (129792-2014-012).

### 4.3. Genome-Wide DNA Methylation Analysis

DNA was extracted from three normal colon (NC) and eight UC patients, as well as one HCT116 cell line. Genome-wide DNA methylation was assessed using the Infinium Human Methylation 450K BeadChip kit (Illumina, San Diego, CA, USA) according to the manufacturer’s instructions. It contained over 480,000 CpGs which covered approximately 2% of the CpGs in the human genome. Preprocessing of raw data was conducted using the GenomeStudio application by Macrogen (Macrogen). Background corrections and dye bias equalizations were performed using methylumi and lumi R packages, respectively [37,38]. Signals were further normalized using the beta-mixture quantile normalization method (BMIQ) [39]. Briefly, CpGs with high quality (*p* < 0.05) were only used for analyses. Each methylation data point was represented by fluorescent signals from the M (methylated) and U (unmethylated) alleles. Background intensity computed from a set of negative controls was subtracted from each data point. The ratio of fluorescent signals was then computed from the two alleles using the beta (β)-value. The β-values of each CpG site ranging from 0 to 1 reflected percentage methylation levels from 0%–100%, respectively. Differentially methylated CpG sites were defined as sites with a *p* value (independent samples *t*-test) <0.05 and a difference of average β-values between UC and N of >0.2. A total of 4820 CpGs were identified. Among them, 237 CpGs were hypermethylated (>1.7-fold increase) in UC samples compared to NC samples.

### 4.4. DNA Methylation Analysis

For methylation analyses, genomic DNA was isolated from 8 normal colon tissues and 79 UC primary tissues using phenol/chloroform. Primer pairs for methylation analyses were preferentially designed for CpG islands of the target genes. For methylation-specific PCR (MSP) and bisulfate sequencing, all primers were designed by MethPrimer (http://www.urogene.org/methprimer). The primer sequences are listed in Table S2. Methylation analyses, including MSP and bisulfite sequencing analyses, were performed as previously described [40].

### 4.5. Quantitative Methylation-Specific PCR (qMSP)

Bisulfite modification of 2 μg genomic DNA was performed using the EZ DNA Methylation kit (Zymo Research, Irvine, CA, USA). For positive and negative controls*,* in vitro methylated DNA (IVD) and H_2_O were used, respectively. For methylation analyses of target genes, quantitative MSP amplification was performed on bisulfite treated samples and normalized using the *Alu* element. All primers were designed using MethPrimer and listed in Table S2. Real-time PCR was performed by a CFX96^TM^ real-time system (Bio-Rad, Hercules, CA, USA).

### 4.6. Quantitative Real-Time RT-PCR (qRT-PCR)

Total RNA was isolated from human normal colon and UC patient tissues using TRI-Solution (BioScience Technology, Rockaway, NJ, USA) following the manufacturer’s protocol. RNA quantity was measured using a NanoDrop 2000/2000c instrument (Thermo Scientific, Waltham, MA, USA) and 1 µg of total RNA was reverse-transcribed into cDNA using the iScript^TM^ cDNA Synthesis kit (BioRad, Hercules, CA, USA). For expression studies, primers were designed using the Primer3 web tool (http://frodo.wi.mit.edu/primer3), and listed in Table S2. Quantitative RT-PCR was performed on a CFX96^TM^ Real-Time PCR Detection System (Bio-Rad) using a Syber Green master mix (Thermo Scientific, Waltham, MA, USA). The expression levels of target genes were normalized by β-actin levels, and all relative quantifications of expressions were calculated using the ΔΔ*C*_t_ method.

### 4.7. Computational Analysis

The CpG islands of target genes were predicted and determined by the University of California Santa Cruz (UCSC, Santa Cruz, CA, USA) genome browser (http://www.genome.ucsc.edu) and the CpG Island Searcher (http://cpgislands.usc.edu), following basic limit values. Signal pathways of target genes were predicted by the Super-pathway category within the GeneCard database (http://www.genecards.org/cgi-bin) that included KEGG (http://www.kegg.jp/kegg/pathway.html). For in silico biological functional analyses of target genes, gene ontology (GO) was analyzed with the GeneMANIA application [41]. The *p* value threshold was limited to 10^−3^.

### 4.8. Statistical Analysis Nel

Quantified data are expressed as the mean ± standard deviation (SD). Significance testing was conducted using the Student’s *t*-test.

### 4.9. Availability of Data and Materials

Raw files of the Infinium Human Methylation 450K array supporting the results of this study are available in the Gene Expression Omnibus (GEO) repository under the accession number GSE81211.

## Figures and Tables

**Table 1:** Basic characteristics of the UC patient samples in this study.

Characteristics	Number (Mean)
Total no. of patients	79
Age (years) median (range)	42.4 (16–68)
**Gender, *n* (%)**	
Male	48 (60.8)
Female	31 (39.2)
**Duration of disease**	
≤1 year	41 (51.9)
1–8 year	24 (30.4)
>9 year	14 (17.7)
**Lesion location, *n* (%)**	
Proctitis	44 (55.7)
Left sided colitis	25 (31.6)
Pancolitis	10 (12.7)
**Mayo endoscopic score, *n* (%)**	
Normal or inactive	3 (3.8)
Mild disease	23 (29.1)
Moderate disease	44 (55.7)
Severe disease	9 (11.4)
**Clinical type, *n* (%)**	
Only one episode	39 (49.4)
Chronic relapsing	35 (44.3)
Chronic continuous	5 (6.3)

## Supplementary Materials

Supplementary materials can be found at www.mdpi.com/1422-0067/17/8/1291/s1.
