# Supplementary material for: A Genome-Wide Methylation Approach Identifies a New Hypermethylated Gene Panel in Ulcerative Colitis
Source: Int J Mol Sci. 2016 Aug 9;17(8):1291. doi: 10.3390/ijms17081291 (PMC5000688; doi:10.3390/ijms17081291)
Supplement: Supplementary file 1 [file ijms-17-01291-s001.pdf]

Supplementary Materials: Genome-Wide Methylation Approach Identifies a New Hypermethylated Gene Panel in Ulcerative Colitis

Keunsoo Kang, Jin-Han Bae, Kyudong Han, Eun Soo Kim, Tae-Oh Kim and Joo Mi Yi

Table S1. List of candidate genes.

Gene	Accession No.	Gene Description	Methylation Fold Changes (UC/N)
ACAT2	NM_005891	Acetyl-CoA acetyltransferase 2	2.0
ACSS2	NM_018677	Acyl-CoA synthetase short-chain family member 2	1.7
CALN1	NM_031468	Calneuron 1	2.2
CDRT15	NM_001007530	CMT1A duplicated region transcript 15	1.8
CDX2	NM_001265	Caudal type homeobox 2	1.8
DNAJC17	NM_018163	DnaJ (Hsp40) homolog, subfamily C, member 17	2.3
ESYT3	NM_031913	Extended synaptotagmin-like protein 3	1.8
EXT1	NM_000127	Exostosin glycosyltransferase 1	1.9
FAM19A5	NM_015381	Family with sequence similarity 19 (chemokine (C-C motif)-like), member A5	2.6
FAM217B	NM_022106	Family with sequence similarity 217, member B	2.3
FAM46A	NM_017633	Family with sequence similarity 46, member A	1.8
FSTL1	NM_007085	Follistatin-like 1	1.7
FXYD3	NM_005971	FXYD domain containing ion transport regulator 3	1.9
GAS1	NM_002048	Growth arrest-specific 1	1.8
H3F3AP4	NR_002315	H3 histone, family 3A, pseudogene 4	1.7
HNMT	NM_006895	Histamine N-methyltransferase	1.9
HNRNPF	NM_004966	Heterogeneous nuclear ribonucleoprotein F	1.9
HOXA9	NM_152739	Homeobox A9	1.8
IFITM1	NM_003641	Interferon induced transmembrane protein 1	1.8
KBTBD11	NM_014867	Kelch repeat and BTB (POZ) domain containing 11	2.0
KIAA1614	NM_020950	KIAA1614	1.9
LHPP	NM_022126	Phospholysine phosphohistidine inorganic pyrophosphate phosphatase	1.7
LHX1	NM_005568	LIM homeobox 1	1.7
MBNL2	NM_144778	Muscleblind-like splicing regulator 2	1.7
MED26	NM_004831	Mediator complex subunit 26	1.7
MIB2	NM_080875	Mindbomb E3 ubiquitin protein ligase 2	1.8
MICAL1	NM_022765	Microtubule associated monooxygenase, calponin and LIM domain containing 1	1.7
MYO3A	NM_017433	Myosin IIIA	2.1
NADK	NM_023018	NAD kinase	1.7
PAX5	NM_016734	Paired box 5	1.9
PITPNC1	NM_012417	Phosphatidylinositol transfer protein, cytoplasmic 1	1.9
PLEC	NM_000445	Plectin	2.4
PLLP	NM_015993	Plasmolipin	1.8
PRICKLE1	NM_153026	Prickle homolog 1 (Drosophila)	1.7
PTGER2	NM_000956	Prostaglandin E receptor 2 (subtype EP2), 53kDa	2.0
RAB3B	NM_002867	RAB3B, member RAS oncogene family	1.7
RIBC2	NM_015653	RIB43A domain with coiled-coils 2	2.1
RPL31	NM_000993	Ribosomal protein L31	1.8
SCAMP1	NM_004866	Secretory carrier membrane protein 1	1.9
SETD1B	NM_015048	SET domain containing 1B	2.0

Table S1. *Cont.*

Gene	Accession No.	Gene Description	Methylation Fold Changes (UC/N)
SLC38A2	NM_018976	Solute carrier family 38, member 2	1.8
SLC38A4	NM_018018	Solute carrier family 38, member 4	1.8
TFAP2E	NM_178548	Transcription factor AP-2 epsilon (activating enhancer binding protein 2 epsilon)	2.4
THSD7A	NM_015204	Thrombospondin, type I, domain containing 7A	1.7
TSPYL1	NM_003309	TSPY-like 1	1.8

Table S2. Primer information in this study.

Gene	Target	Methylation Specific PCR	
		**Forward Primer (5′–3′)**	**Reverse Primer (5′–3′)**
ACAT2	Unmehtylation	TTTTTGTGATGTTAAGATGTTTTTG	AATCCTATACATTTCTCCTAACACC
	Methylation	ATTTTTGTGACGTTAAGACGTTTTC	AAAATCCTATACGTTTCTCCTAACG
ACSS2	Unmehtylation	TTAGGATTGTGTAATTAGATTATGG	ATAAAATAATAAAAAATAACCCAAC
	Methylation	TTAGGATTGTGTAATTAGATTACGG	CATAAAATAATAAAAAATAACCCGAC
CALN1	Unmehtylation	TTTGGTTATTGGTTTTTTTATTTTG	AAAAACTACCCACATTAACTCCAAC
	Methylation	TTTCGGTTATTGGTTTTTTTATTTC	AACTACCCACGTTAACTCCGAC
CDX2	Unmehtylation	ATAGAGTTTTGTAAATATTTGTTAATTATG	CCTTCCCACTAAACTACAAAAACAA
	Methylation	GAGTTTTGTAAATATTCGTTAATTACGG	CTTCCCACTAAACTACAAAAACGAA
DNAJC17	Unmehtylation	GTTGTAAGATTAAGTTTTTGGATGT	ATCTCAACTCTACAAACCCTACAAT
	Methylation	GTCGTAAGATTAAGTTTTTGGACGT	ATCTCAACTCTACAAACCCTACGAT
ESYT3	Unmehtylation	GGGTGTTTTATAGATGGTTAAGATG	ACTAACCCCTAAAACCTACAACACT
	Methylation	GGGCGTTTTATAGATGGTTAAGAC	AACTAACCCCTAAAACCTACAACG
EXT1	Unmehtylation	GTTTAGGAGGTTAGATTTTTTAGGGT	TACAAACTACCAACAAACTACCAAA
	Methylation	TTTAGGAGGTTAGATTTTTTAGGGC	AAACTACCGACGAACTACCG
FAM19A5	Unmehtylation	GGATTAGGTAGTGGTAGAGGTGTTT	ATATACCCTATTACCCAAAATCAAA
	Methylation	GGATTAGGTAGTGGTAGAGGTGTTC	ATATACCCTATTACCCAAAATCGAA
FAM217B	Unmehtylation	GTAGGGATGAGAGATGATTTTTTGA	AATAACAAACCAAAAATAAAACACA
	Methylation	GTAGGGATGAGAGACGATTTTTC	AAAATAACGAACCAAAAATAAAACG
FAM46A	Unmehtylation	AGAGAATTTGTAGTAAAGGTATTGG	AAACTAAACAAACCAAATAAACACA
	Methylation	AGAGAATTCGTAGTAAAGGTATCGG	AAAACTAAACGAACCGAATAAACG
FSTL1	Unmehtylation	TATTTTTTTGTGGTAGGGATTTATG	CTCCCCCTTCTACTACAACAAC
	Methylation	TTATTTTTTTGTGGTAGGGATTTAC	CTCCCCCTTCTACTACAACGAC
GAS1	Unmehtylation	TTTTTAAGAGTAGGAGGAGTTTTGG	CAAACCACTAAAATAAAATTACAAA
	Methylation	TTTTTTAAGAGTAGGAGGAGTTTCG	TACGAACCGCTAAAATAAAATTACG
H3F3AP4	Unmehtylation	TATATATTGTTTAGAGGTTGATGTGA	ACCAAATACAACCAAATCCTACAAA
	Methylation	TTATATATTGTTTAGAGGTCGACGC	AAATACGACCGAATCCTACGAA
HNRNPF	Unmehtylation	GAGGTTTGGGGGTTTTTTATATTAT	CCTAACAACAAAACAAAACTACATC
	Methylation	GAGGTTTGGGGGTTTTTTATATTAC	CTAACGACAAAACGAAACTACGTC

Table S2. *Cont.*

Gene	Target	Methylation Specific PCR	
		Forward Primer (5′–3′)	Reverse Primer (5′–3′)
HOXA9	Unmehtylation	TAAATTTTATTGTAGAGTGGTATGA	AATCACCTAATAAATTACCAACACC
	Methylation	ATAAATTTTATCGTAGAGCGGTACG	ACAATCACCTAATAAATTACCGACG
KBTBD11	Unmehtylation	TTTGGTATTTATGGAAATTTTATTGT	ATATAACCTCTACCTCCCACTCAAA
	Methylation	TTTCGGTATTTATGGAAATTTTATC	AATATAACCTCTACCTCCCACTCG
KIAA1614	Unmehtylation	AGGTTTGGTTTTTTTAGTTTTTTTT	TAACATCAACTATCCATTCACAAC
	Methylation	AGGTTCGGTTTTTTTAGTTTTTTTC	AATAACATCAACTATCCGTTCACG
LHPP	Unmehtylation	AGTAGTTGGGATTATAGGTATGTGT	AATCACTTAAAATCAAAAATTCAAA
	Methylation	AGTAGTTGGGATTATAGGTATGCGT	TAAATCGCTTAAAATCAAAAATTCG
LHX1	Unmehtylation	GTTGTGATTGGAGTTATGATGTATG	CCTCAACCCTTAACCTACCTAACA
	Methylation	AGTTGTGATTGGAGTTACGATGTAC	TCGACCCTTAACCTACCTAACG
MIB2	Unmehtylation	AGTTTGGGAGTTAATAGGATTATGT	CCATTTTAACCAAACTAATTTCAAA
	Methylation	AGTTTGGGAGTTAATAGGATTACGT	ACCATTTTAACCAAACTAATTTCGA
MICAL1	Unmehtylation	TAAATTTTTTGAGGAGTTTGAGTGT	CATACAAAACTAACTCTTTCCCCAC
	Methylation	TTTAAATTTTTTGAGGAGTTTGAGC	GTACAAAACTAACTCTTTCCCCG
MED26	Unmehtylation	TTTTTAATTATGGGGTTTTTTGTTG	CCTCAACACCACTAACATTTAAACA
	Methylation	TTTTTAATTACGGGGTTTTTTGTC	CTCGACACCACTAACATTTAAACG
MYO3A	Unmehtylation	AATTTATTATATTTTTTGGGTTTGA	ACAAAACCCTTAACTAACTACCAAA
	Methylation	AATTTATTATATTTTTCGGGTTCGA	GCAAAACCCTTAACTAACTACCGA
NADK	Unmehtylation	TAGTTTGTGGGGTGTGTAAAATATG	TATCCATCAAAACTAAAAAAACAAC
	Methylation	TTTGTGGGGTGCGTAAAATAC	TATCCGTCGAAACTAAAAAAACG
PAX5	Unmehtylation	TTTTTTAAAAGTATTTGTTTGGTTGA	AATCACAACCCTCTACACTATACAC
	Methylation	TTTTTAAAAGTATTTGTTTGGTCGA	GCGACCCTCTACGCTATACG
PITPNC1	Unmehtylation	GTATTTAGGGTATAATGGAGAGTGG	AAACAACATATCTCTACCAAACAACAA
	Methylation	AGTATTTAGGGTATAATGGAGAGCG	AACATATCTCTACCAAACGACGAA
PLEC	Unmehtylation	TTGTTTTTTAGGTTTTTAGTTTTGT	CTAACCCCTAATAATAAAATCAAC
	Methylation	TTGTTTTTTAGGTTTTTAGTTTCGT	ACTAACCCCTAATAATAAAATCGAC
PLLP	Unmehtylation	GTGTTTGGAGTTATATAGGGATTTG	TACTAACTTTCAACAAAAACTCAAC
	Methylation	GCGTTTGGAGTTATATAGGGATTC	TACTAACTTTCGACGAAAACTCGA

Table S2. *Cont.*

Gene	Target	Methylation Specific PCR	
		**Forward Primer (5′–3′)**	**Reverse Primer (5′–3′)**
PRICKLE1	Unmehtylation	TAGATTAGTTTGTGGGATTATATGA	CTATTTAAATTCCTTCTCCTTCAAA
	Methylation	TAGATTAGTTTGTGGGATTATACGA	CTATTTAAATTCCTTCTCCTTCGAA
PTGER2	Unmehtylation	TTTTGATTTTTTGTGTATTTTGTGT	CAATCTAACCTCAACTAAAAAACATT
	Methylation	GTTTCGATTTTTTGTGTATTTTGC	AATCTAACCTCGACTAAAAAACGTT
RAB3B	Unmehtylation	TTTTTTTGGGTTTTTGTGAAGAT	CCCCCATATCTAAATAACTCTCATA
	Methylation	TTTTTTTGGGTTTTTGTGAAGAC	CCCCCATATCTAAATAACTCTCGTA
RIBC2	Unmehtylation	TAGTTATTAGAGTTGTTGGGGTTGA	CACTTAATAACACAAATAAAACATA
	Methylation	TTTAGTTATTAGAGTCGTTGGGGTC	GCTTAATAACGCGAATAAAACGTA
RPL31	Unmehtylation	TTATTTATGTATAGGGATGATGTGA	CCAACACTAACTAAACCTACATCAAA
	Methylation	TTATTTACGTATAGGGACGACGC	CAACACTAACTAAACCTACGTCGAA
SCAMP1	Unmehtylation	GGGTTTTATATTTTTTGAGGATTTTG	CCACACTCCAAACTTTAACTACAAC
	Methylation	GGGTTTTATATTTTTCGAGGATTTC	ACGCTCCGAACTTTAACTACG
SETD1B	Unmehtylation	ATTTTTTTGGAAGGGTTATTTTTTT	TAAAAACCAACAAATTATTATCATC
	Methylation	TTTTTCGGAAGGGTTATTTTTTC	TAAAAACCAACAAATTATTATCGTC
SLC38A2	Methylation	TAAGGGAGTGAGTTTTGTTTTTTTT	ACAAATCTAAAACACTTTCTACAAA
	Unmehtylation	TAAGGGAGTGAGTTTTGTTTTTTTC	ATACGAATCTAAAACGCTTTCTACG
SLC38A4	Methylation	TTTAATAATATTGGGTATATTTGTGG	CCAACATTACAAACAAAACAACAAC
	Unmehtylation	TTTAATAATATTGGGTATATTCGCG	CAACATTACAAACAAAACAACGAC
TFAP2E	Methylation	TTTAGTTTAGGAAGTTTGAGAGTGA	CATACACACACAATATCAATACATA
	Methylation	TTTTTAGTTTAGGAAGTTTGAGAGC	CATACGCACACAATATCAATACGTA
THSD7A	Unmehtylation	TTTTTTTGTTTTTTTGGGTTTATGT	ACCTCTAACAAAAATACTACTACCACT
	Methylation	GTTTTTTTTGTTTTTTTGGGTTTAC	GCCTCTAACGAAAATACTACTACCG
TSPYL1	Unmehtylation	TATTGGAGAGTAGGTATTGTTATGG	TACTAAATCTAAAAACACAAACAAC
	Methylation	TATTGGAGAGTAGGTATTGTTACGG	TACTAAATCTAAAAACACGAACGAC
FOXE1	Unmehtylation	TCGGTTTTTGAATTTTTCGGGTTTTAGTCGATC	GATAAAAACCCCACAACGTCAAAACG
	Methylation	GTTGGTTTTTGAATTTTTTGGGTTTTAGTTGATTG	AACAACACAATAAAAACCCCACAACATCAAAACA
SYNE1	Unmehtylation	GTGGTTGGGTTTTTGTAGTTTTGTAGATTGTG	CAACTCTCTACACCCAAACTCAACA
	Methylation	GTTGGGTTTTCGTAGTTTTGTAGATCGC	CTACGCCCAAACTCGACG
Alu	Methylation	ATTAGTCGGGCGTGGTGG	CCCGAATTCAAACGATTCTCC

Table S2. *Cont.*

Gene	Locus	Bisulfite Sequencing	
		**Forward Primer (5′-3′)**	**Reverse Primer (5′-3′)**
FAM217B	chr20 (58,515,279-58,515,559)	GGTTCGCGGTTTTAGGGTTA	AACGAACCAAAAATAAAACGCAC
KIA1614	chr1 (180,881,647-180,882,085)	GAGGTTYGGTTTTTTTAGTTTTTTT	AAAAAAAATAACATCAACTATCC
RIBC2	chr22 (45,809,169-45,809,433)	TAGGGGTTGGGTTAGGGTTA	CTACTACTTATAAAAAATTTCAAATC
**Gene**		**Quantitative Real-Time RT-PCR**	
		**Forward Primer (5′-3′)**	**Reverse Primer (5′-3′)**
FAM217B		TGGACGATGTTAGTTAATGATAAATC	TTAAACTCCAACTCTAAAAACCGTA
KIA1614		AGTGTTAAGGGGTTAGGATTAATTC	AACGACCCGAAACTTTTATACG
RIBC2		GTTAATTTTTAGGGATCGGGTAGAC	GAACTTTAAATCCTAATTCCTCGCT
ACTB		AGCGAGCATCCCCCAAAGTT	GGGCACGAAGGCTCATCATT

	Colon cancer cell line										
Gene	CACO2	COLO320	COVO	DLD1	HT29	RKO	SW480	SW620	HCT116	DKO	IVD
FAM217B	M	U	M	M	M	M	M	M	M	U	M
KIAA1614	M	M	M	M	M	M	M	M	M	U	M
RIBC2	U	M	M	M	M	M	M	M	M	U	M

Figure S1. The best candidate genes are frequently hypermethylated in most of colon cancer cell lines. Summary of the methylation patterns for FAM217B, KIA1614, and RIBC2 gene in 10 colon cancer cell lines by MSP analysis. M and U indicates methylation and ummethylation, respectively. DKO and IVD were used for unmethylation and methylation controls in MSP analysis.

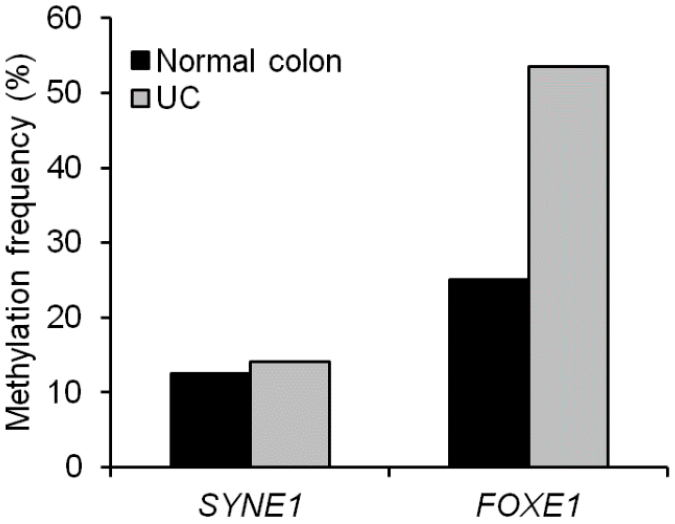

Figure S2. Methylation frequency of *SYNE1* and *FOXE1* in UC patients. MSP was performed in 71 UC samples and 8 normal colon tissues. SYNE1 and FOXE1 showed 14.1% (10 out of 71) and 53.5% (38 out of 71) methylation frequencies, respectively. Primer set for MSP analysis was used by Papadia et al. (2014).

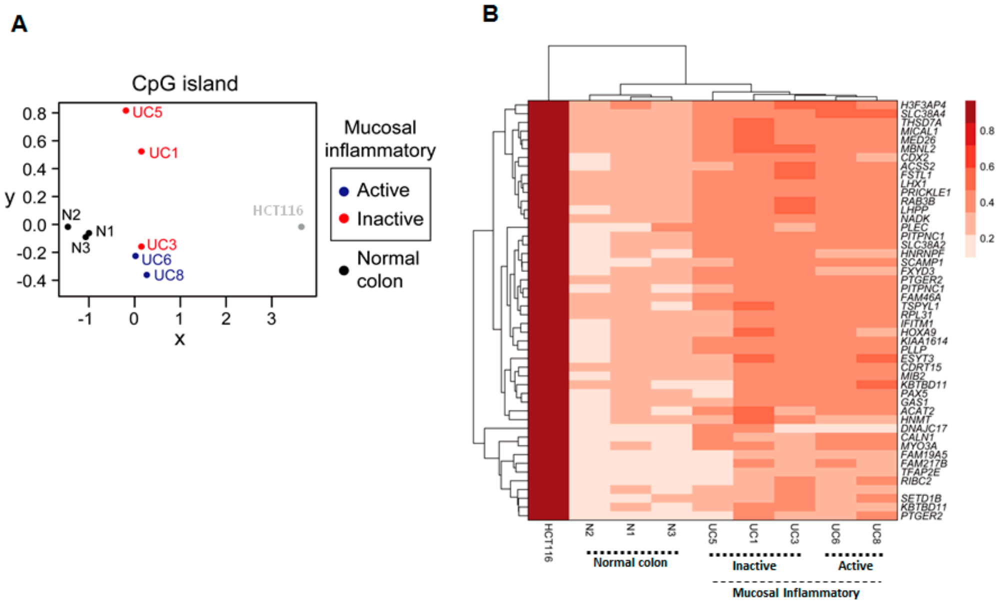

Figure S3. DNA methylation profile is associated to the inflammatory activity. (**A**) Multidimensional scaling (MDS) was used to estimate the relationship between samples. The MDS plot shows the similarity between individual samples according to methylation status of the 48 probes. The x and y axis represent arbitrary numbers; (**B**) Clinical implication of the hypermethylated CpG sites. Unsupervised hierarchical clustering was performed with β-values of the CpG sites. Samples were matched with the severity of inflammatory activity.
